# Radiation-induced osteoradionecrosis of the ribs in a patient with breast cancer: A case report^☆^

**DOI:** 10.1016/j.radcr.2022.01.067

**Published:** 2022-06-13

**Authors:** Suhong Kim, Young Seon Kim

**Affiliations:** a Graduate School of Medical Science and Engineering, Korea Advanced Institute of Science and Technology (KAIST), Daejeon, Republic of Korea; b Korea Advanced Institute of Science and Technology (KAIST), KI for Health Science Technology, Daejeon, Republic of Korea; c Department of Radiology, Yeungnam University College of Medicine, Daegu, Republic of Korea

**Keywords:** Osteoradionecrosis, Breast neoplasms, Rib, Radiation, Complication

## Abstract

Osteoradionecrosis of the chest wall after radiation therapy for breast cancer is rare; however, it is one of the most severe complications of radiation treatment. Radiologically, osteoradionecrosis can manifest as a focal lucent area in bone, periostitis, sclerosis, and cortical irregularity of bones on X-ray or computed tomography; therefore, differentiation from bone metastasis can be challenging. Associated insufficiency fractures, ulceration, and skin necrosis may also occur. We encountered a patient with osteoradionecrosis in the left anterior ribs after radiation therapy for breast cancer. Chest computed tomography revealed cortical irregularity with severe sclerotic changes of the anterior arc of the left fist to the fourth ribs. The patient's skin on the left chest wall exhibited ulceration with purulent discharge. Ultrasonography of the left chest wall revealed diffuse skin thickening with hyperechoic changes in the subcutaneous fat layer of the left chest wall with calcifications. The patient underwent rib resection and chest wall reconstruction. Recognizing characteristic imaging features of osteoradionecrosis is important for radiologists to differentiate it from bone metastasis and plan appropriate treatment.

## Introduction

Breast cancer is the most common malignant neoplasm among women worldwide and is the major cause of cancer-related mortality in most countries [1]. The primary treatment methods for breast cancer include surgery (breast-conserving surgery or mastectomy), chemotherapy, endocrine therapy, and radiation therapy. Radiation therapy is commonly combined with other treatment methods; therefore, many breast cancer patients also undergo radiation treatment. External beam radiation is an established treatment modality for breast cancer, the primary goal of which is the eradication of residual cancer cells; however, irradiation also affects normal cells in the operative bed as well as adjacent structures [2]. Early complications occurring within weeks to months after completion of radiation therapy include breast edema, fat necrosis, radiation-induced pneumonia, and pleural effusion [2]. Complications that appear even later include breast fibrosis, pulmonary fibrosis, cardiomyopathy, and radiation-induced malignancies [2]. Although osteoradionecrosis (ORN) of the chest wall after radiation therapy for breast cancer is rare, it is one of the most serious complications of radiation treatment. Vascular compromise caused by radiation damage to blood vessels can affect osteoblasts and osteoclasts and may be a possible cause of ORN [2]. Radiologically, ORN can manifest as a focal lucent area in bone, periostitis, sclerosis, and cortical irregularity of bones on X-ray or computed tomography (CT); therefore, differentiation from bone metastasis can be challenging. Associated insufficiency fractures, ulceration, full-thickness skin necrosis, and superimposed infection of the soft tissue and bone may be present [3,4]. As such, recognizing the clinical and imaging features of ORN is very important for radiologists to differentiate it from bone metastasis and plan appropriate treatment.

## Case report

A 79-year-old woman presented with a painful open wound in the left anterior chest wall. Four months earlier, a scab appeared on her left chest area and fell off, and pus started to emerge 1 month previously. She underwent a modified radical mastectomy with axillary lymph node dissection for left breast cancer 25 years previously, followed by radiation therapy. Her clinical records documented that radiation therapy consisted of 6000 cGy for 6.5 weeks to the left chest wall (operative bed), 5000 cGy for 5 weeks to the supraclavicular fossa and axilla, and 4500 cGy for 5 weeks to the internal mammary lymph node chain. She underwent adjuvant chemotherapy with 6 cycles of 5-fluorouracil, epirubicin, and cyclophosphamide. On physical examination, an ulceration measuring approximately 1 cm in diameter with purulent discharge was observed in her left chest wall.

**Fig. 1:**
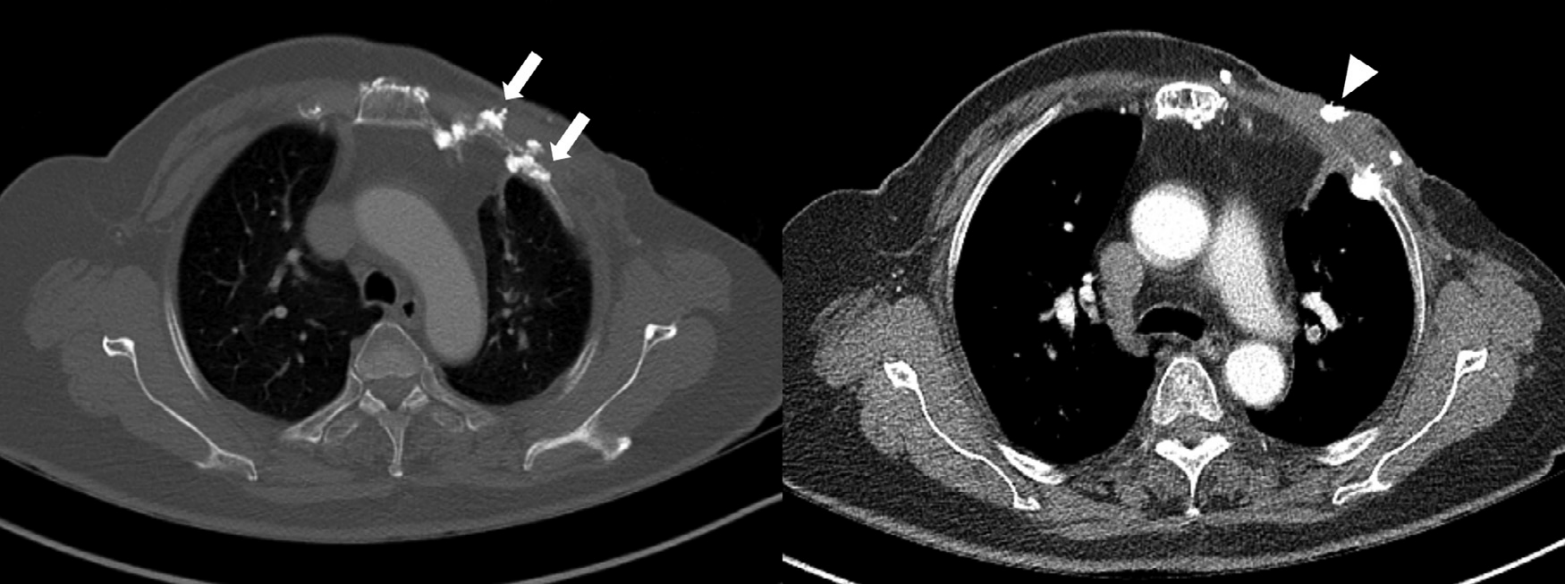
Radiological findings of osteoradionecrosis of the left anterior ribs in a 79-year-old woman. (A) Computed tomography (CT) of the chest wall revealing fractures with severe sclerotic change and cortical irregularity of left first to fourth ribs (arrows). Ulceration at overlying skin layer is also noted (arrowhead).

**Fig. 2:**
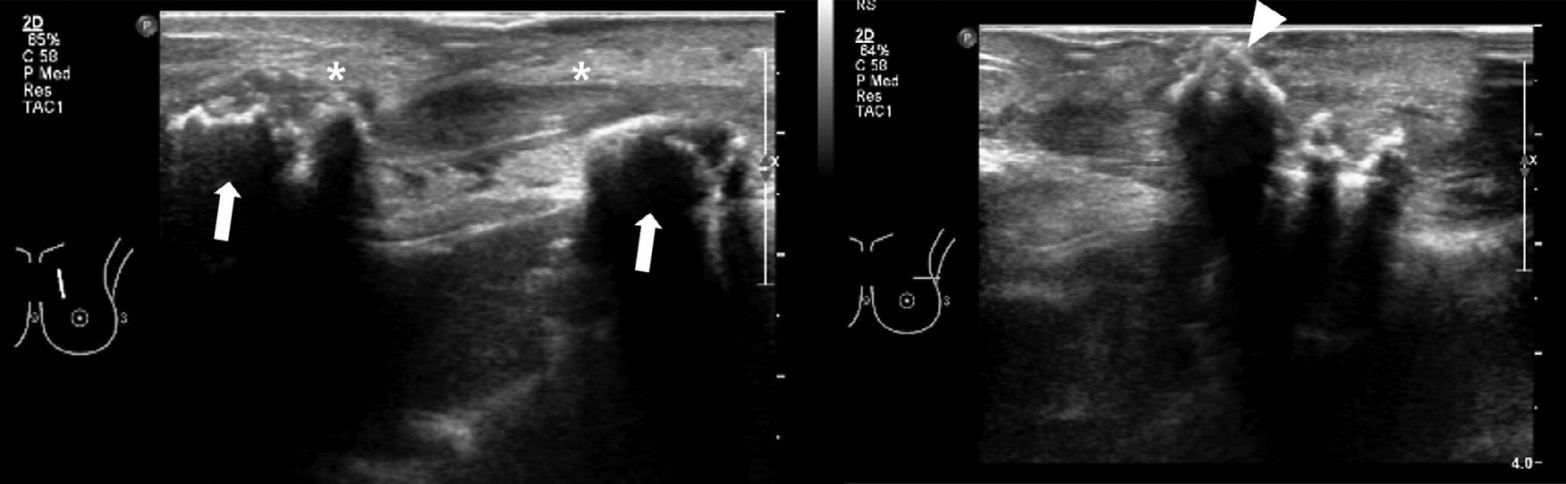
Radiological findings of osteoradionecrosis of the left anterior ribs in a 79-year-old woman. (B) Ultrasonography of the left chest wall revealing diffuse thickening of the skin and edematous change of subcutaneous fat layer. Calcifications within the subcutaneous fat layer is noted (arrowhead).

**Fig. 3:**
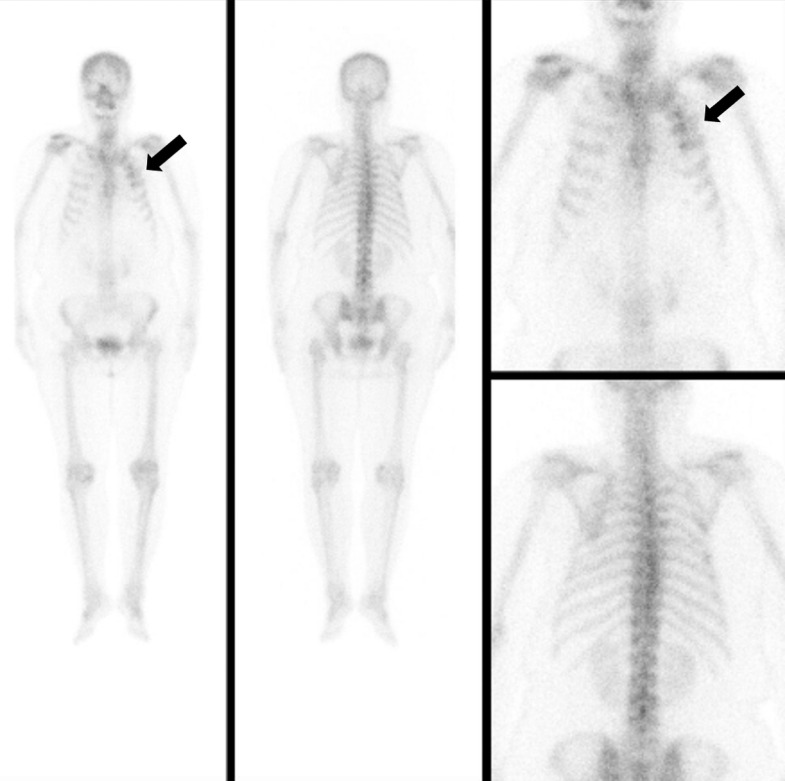
Radiological findings of osteoradionecrosis of the left anterior ribs in a 79-year-old woman. (C) Whole body bone scan exhibiting hot uptake in the left first to fourth ribs, suggesting fractures (arrows).

Contrast-enhanced CT of the chest revealed severe sclerotic changes with cortical irregularity and discontinuity of the anterior arcs of the left first to fourth ribs and destroyed costal cartilage (Fig. 1). Multiple calcifications within the pectoralis muscle and subcutaneous fat layer were observed. Focal ulcerations were also observed in the overlying skin layer. Ultrasonography of the left chest wall revealed diffuse skin thickening and increased echogenicity of the subcutaneous fat layer (Fig. 2). Calcification was also observed at the base of skin ulceration. A whole-body bone scan revealed hot uptake in the left first to fourth ribs, suggesting fractures (Fig. 3). There were no other uptake lesions visible on the whole-body bone scan. Thus, considering the clinical manifestations, and imaging findings, the patient was diagnosed with ORN. Partial resection of the left first to fourth ribs and costal cartilage, part of the sternum and body, sternal end of the left clavicle, and wide debridement and irrigation were performed. Bone biopsy confirmed osteomyelitis in the left first to fourth ribs, and the patient was definitively diagnosed with ORN accompanied by cellulitis and abscess. After surgery, the wound did not heal well; as such, 4 additional surgeries (curettage and irrigation) were performed. Finally, she underwent chest wall reconstructive surgery with pedicled latissimus dorsi muscle flap and meshed skin graft from her left thigh to cover an open wound measuring approximately 30 × 20 cm and was discharged.

**Fig. 4:**
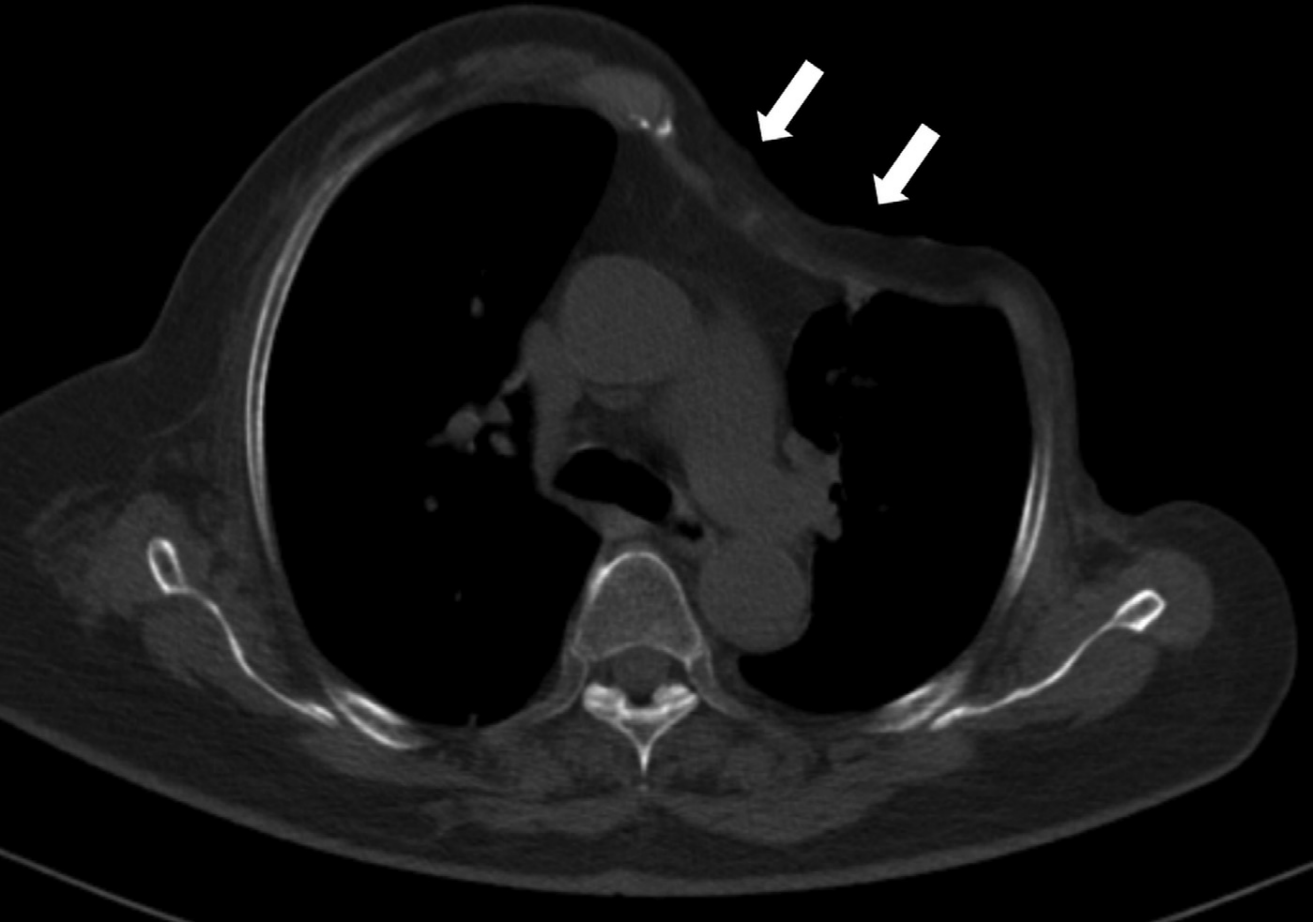
Radiological findings of osteoradionecrosis of the left anterior ribs in a 79-year-old woman. (D) Follow-up chest CT performed 7 months after reconstruction surgery revealed deformed left chest wall (arrows) without recurrence of ulceration or infection.

No complications or ulcer recurrence were evident 13 months after surgery (Fig. 4).

## Discussion

Radiation therapy is a primary treatment option for cancers and is used in both curative and palliative therapies for most solid tumors [5]. For the treatment of breast cancer, radiation therapy is one of the most important and commonly used treatment options in combination with surgery, and chemotherapy. Although highly effective in controlling microscopic residual lesions in the operative bed, and despite its many advantages, many early or delayed complications can occur. During the weeks to months after completion of radiation therapy, radiation dermatitis, breast edema, fat necrosis, radiation-induced pneumonia, and pleural effusion may occur [2,5]. Moreover, months to years after the completion of radiation therapy, soft tissue fibrosis, brachial plexopathy, lymphedema, bone fracture, and pulmonary fibrosis can occur [2,5]. After more than 10 years, cardiomyopathy, and radiation-induced malignancies can occur [2].

An acute side effect of radiation therapy related to bone is hematological suppression and inflammation. Late skeletal side effects of radiation therapy include demineralization and osteoporosis and deformities in children [5]. Demineralization and osteoporosis may increase the risk for insufficiency fractures, especially of the ribs, femur, and pelvis [5]. Most patients are treated and healed with conservative management.

Radiation-induced rib fracture is a rare complication of radiation therapy for breast and lung cancers, with an incidence of 0.1%-0.5% reported in previous studies [6,7]. Severe ORN of the ribs is extremely rare, with very few reports describing radiological findings of ORN [6,8,9]. Focal lucent area in bone, periostitis, sclerotic change, cortical irregularity, and insufficiency fractures can be observed on radiography or CT [6]. In the early stage, ORN lesions exhibit decreased uptake of radioisotope on bone scan and, later, increased uptake appears with accompanying fractures. Alhilali et al. reported that ORN may exhibit false-positive findings on positron emission tomography (PET)/CT, which is relatively unreliable in differentiating ORN from bone metastasis [10]. Instead of PET/CT, some CT findings of ORN are helpful in differentiating it from bone metastasis. For bone metastasis, the presence of bony changes with an associated solid or cystic mass is an important clue for diagnosis [10]. Alhilali, et al. analyzed 63 patients (46 with ORN and 17 with tumor recurrence) who were treated with radiation therapy for head and neck cancer [10]. They categorized the pattern of trabecular loss as permeative (<75% loss of trabecular) or lucent (>75%). A permeative pattern was more commonly observed in ORN, and a lucent pattern was more often observed in metastasis. Because radiation damages osteoblasts and osteoclasts, as well as the supply to blood vessels, it leads to a decreased ability to remodel tissue damage. However, in this situation, relatively less bone loss occurs than in the situation in which bone is actively destroyed by bone metastasis. In addition, irradiated bone is susceptible to infection, and intraosseous gas may be observed due to superimposed osteomyelitis in ORN but not in bone metastasis [10]. These imaging findings may be helpful for radiologists in differentiating ORN from bone metastasis.

Clinically, pain, infection, and pathologic fractures are common symptoms of ORN. ORN can be diagnosed by comprehensively considering clinical features, and imaging and pathologic findings. Conservative treatment, including hyperbaric oxygen therapy, may improve radiation-induced tissue injury [11]. ORN with osteomyelitis recurs often, and wound healing is not satisfactory due to radiation-induced fibrotic tissue; therefore, extensive surgical debridement with chest wall reconstruction is important in treatment [3,4,8,12]. However, treatment guidelines for ORN without complications, such as osteomyelitis, have not yet been clearly established.

In conclusion, we reported a case of ORN affecting the anterior arc of the left first to fourth ribs following radiation therapy after mastectomy in a patient with breast cancer. Recognizing the clinical characteristics and correctly interpreting imaging findings will help radiologists to differentiate ORN from bone metastasis and plan appropriate treatment.

## Human and animal rights

All the methods in the study involving human participants were performed in accordance with relevant guidelines and regulations of the institutional and/or national research committee and with the 1964 Helsinki declaration and its lateral amendments or comparable ethical standards.

## Availability of data and materials

The data used for this study, though not available in a public repository, will be made available to other researchers upon reasonable request.

## Funding

This research was supported by the National Research Foundation of Korea (NRF-2021R1G1A1007686).

## Ethical approval

This retrospective study was approved by the Institutional Review Board of Yeungnam University Hospital (IRB file No. 2021-02-055).

## Patient consent

Informed consent has been obtained from the patient for the publication of this case report.
